# High-throughput live-imaging of embryos in microwell arrays using a modular specimen mounting system

**DOI:** 10.1242/bio.031260

**Published:** 2018-04-30

**Authors:** Seth Donoughe, Chiyoung Kim, Cassandra G. Extavour

**Affiliations:** 1 Department of Organismic and Evolutionary Biology, Harvard University, Cambridge MA 02138, USA; 2 Department of Molecular and Cellular Biology, Harvard University, Cambridge MA 02138, USA

**Keywords:** Embryogenesis, Microscopy, High-throughput, Time lapse, Image analysis, Quantitative imaging, Development

## Abstract

High-throughput live-imaging of embryos is an essential technique in developmental biology, but it is difficult and costly to mount and image embryos in consistent conditions. Here, we present OMMAwell, a simple, reusable device to easily mount dozens of embryos in arrays of agarose microwells with customizable dimensions and spacing. OMMAwell can be configured to mount specimens for upright or inverted microscopes, and includes a reservoir to hold live-imaging medium to maintain constant moisture and osmolarity of specimens during time-lapse imaging. All device components can be fabricated by cutting pieces from a sheet of acrylic using a laser cutter or by making them with a 3D printer. We demonstrate how to design a custom mold and use it to live-image dozens of embryos at a time. We include descriptions, schematics, and design files for 13 additional molds for nine animal species, including most major traditional laboratory models and a number of emerging model systems. Finally, we provide instructions for researchers to customize OMMAwell inserts for embryos or tissues not described herein.

## Abstract (precis)

**Summary:** This Methods & Techniques article describes a customizable device for mounting and live-imaging a wide range of tissues and species; complete design files and instructions for assembly are included.

## Introduction

Live-imaging embryos and small organisms in a repeatable, high-throughput manner is crucial for understanding the cellular dynamics that underlie the development of multicellular bodies (Farhadifar et al., 2015; Kuntz and Eisen, 2014). High-throughput imaging allows one to assess subtle phenotypes that can arise from functional genetics experiments, study standing variation within a population, and understand the role of noise in developmental processes. To that end, some research groups have turned to microfluidic devices (Chronis, 2010; Crane et al., 2010; Cornaglia et al., 2015; Wielhouwer et al., 2011). Such microfluidic apparatuses can be constructed to perform precise and complex experimental manipulations, but designing and fabricating these devices is a laborious process. For the purpose of imaging embryos, another option is to fabricate a custom mold that can be used to cast an agar or agarose microwell array. Molds can be milled from plastic (F. Kainz, Notch and FGF signalling in *Gryllus bimaculatus* and their role in segmentation, PhD thesis, Harvard University, 2009) or aluminum (Herrgen et al., 2009), or 3D-printed (Alessandri et al., 2017; Gregory and Veeman, 2013; Wittbrodt et al., 2014). Although these techniques are effective, each was designed to serve the specific needs of one particular species, and therefore it is not straightforward to adapt the existing tools to a new study species.

To address this outstanding need, we developed OMMAwell (Open Modular Mold for Agarose Microwells), an all-in-one device that allows the user to swap out any number of customized mold inserts. These inserts can be prototyped quickly and cheaply, requiring only a laser cutter or a 3D printer. These mold inserts lock into the device, which can be configured in several ways to mount specimens for any upright or inverted microscope that can accommodate a 35 mm petri dish. Using this tool, we can mount dozens of embryos at once in a microwell agarose array, keeping track of each embryo by its position in the array, and then efficiently image them. The modular mold inserts can be exchanged to alter the size, shape, orientation, and spacing of microwells. OMMAwell is therefore adaptable for different experimental designs or even diverse species.

As an example case, we demonstrate a workflow for making a custom mold insert for embryos of the cricket *Gryllus bimaculatus*. These cricket embryos can be imaged through their transparent eggshells. During previous efforts to live-image embryonic development within the eggs – using confocal and widefield microscopy – only a few embryos could be imaged at a time, and the mounting process was inconsistent and time-intensive (Donoughe and Extavour, 2016; Nakamura et al., 2010). Eggs were either manually glued to a coverslip one at a time (Nakamura et al., 2010) or placed in blocks of rubber polymer in which troughs had been hand-cut with a razor (Donoughe and Extavour, 2016). Mounting is similarly laborious for most animal laboratory models, which limits the sample size of experiments and reduces reproducibility. However, we show that with OMMAwell, it is straightforward to mount dozens of embryos in a manner that is suitable for 2D or 3D long-term time-lapse recordings.

In the Supplemental Information, we include detailed instructions for assembling the OMMAwell mounting device, and suggestions for modifying the device to suit the particular requirements of any desired model system. We have also designed and beta-tested mold inserts for embryos of eight additional species, including zebrafish, fruit fly, frog, annelid worm, amphipod crustacean, red flour beetle, and three-banded panther worm, as well as mouse neurospheres. Descriptions, schematics, and design files for all of these mold inserts are provided.

## Results and Discussion

**Fig. 1.**
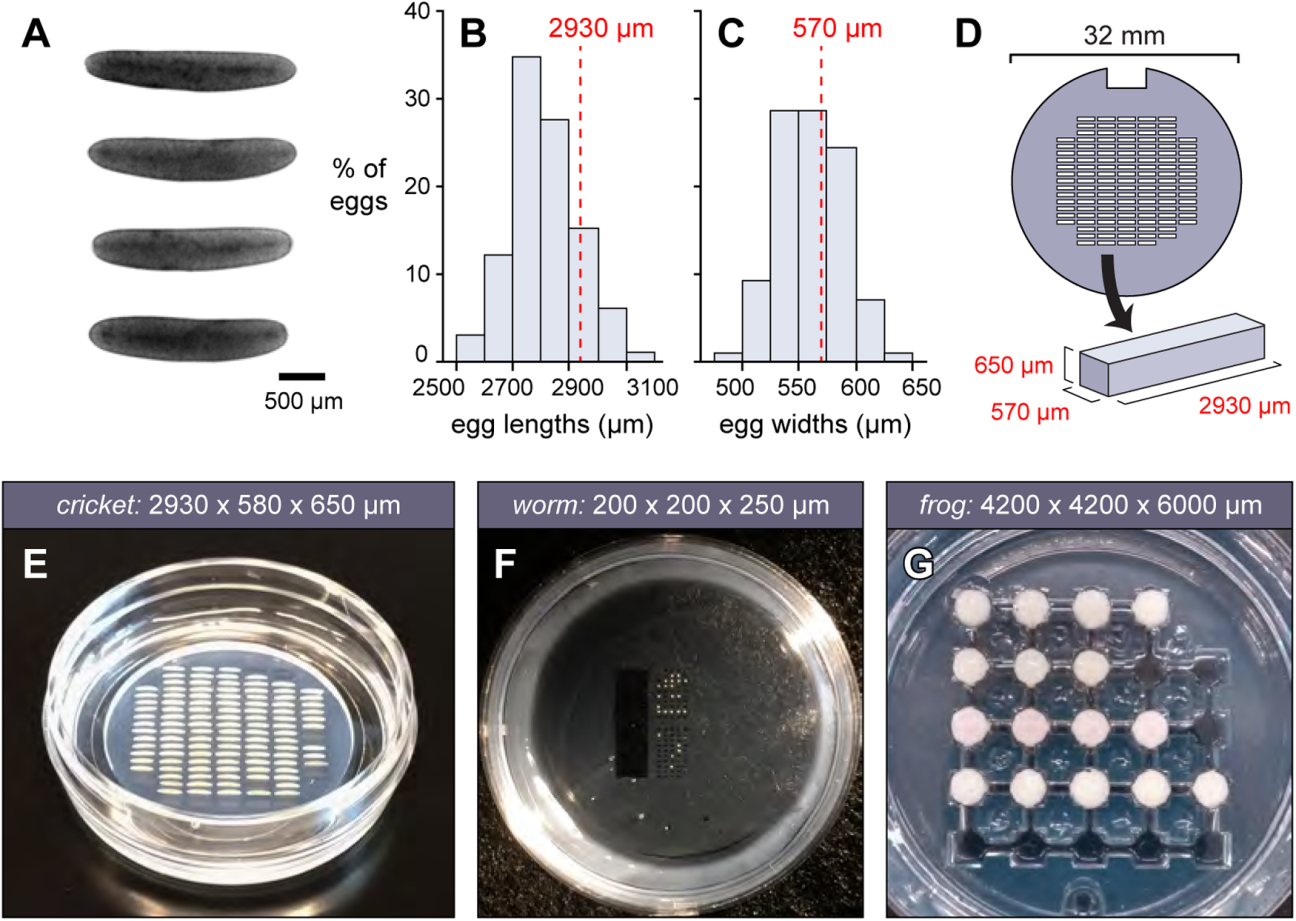
**Designing microwells to hold cricket eggs.** (A) Freshly laid cricket eggs were measured and their (B) lengths and (C) widths plotted (*n*=98; scale bar: 500 µm). Based on the size distribution, we chose dimensions of 2930×570×650 µm (embryo length×width×height), which were values such that approximately 75% of eggs would fit into the troughs. In practice, because the wells are made of agarose, more than 95% of eggs fit into these wells. (D) Raised posts of those dimensions were formed by engraving an acrylic insert. 120 such posts were arranged in a grid. (E) Agarose microwells made using this insert, loaded with cricket eggs. (F,G) Example molds for an annelid worm and coqui frog, with microwell dimensions listed. Photos in F and G by Elaine Seaver (The Whitney Laboratory for Marine Bioscience) and Mara Laslo (Harvard University) respectively.

To design the first iteration of a cricket embryo mold, we collected and measured dimensions of freshly laid eggs (Fig. 1A). The eggs are roughly ellipsoidal in shape, 2500–3200 µm in length, and 475–650 µm in width (Fig. 1B,C). We designed the mold insert to have rectangular posts 2930 µm long by 570 µm wide, each of which will create an agarose microwell able to snugly accommodate the majority of eggs (Fig. 1D).

The ‘mold insert’ is the only piece of the OMMAwell that must be tailored to create wells of appropriate dimensions for one's samples of interest. To make the cricket mold insert, the inverse of our desired pattern was laser-engraved into acrylic to a depth of 650 µm. This is deep enough to contain the embryo, but close enough to the surface to be imaged within the working distance of 5× and 10× microscope objectives. The microwells were arranged into a truncated grid pattern that fit within a 26 mm circle, so that all microwells could be viewed through the circular 27 mm in diameter coverslip (surface area 531 mm^2^) of a 35 mm glass-bottom petri dish (Fig. 1D,E; see the Supplemental Information). Given the dimensions of these particular embryos, we were able to fit 120 wells into the grid. For embryos of different dimensions, more or fewer wells may be able to fit into the coverslip field (e.g. 24 wells for the coquí frog *Eleutherodactylus coqui*; 294 wells for the fruit fly *Drosophila melanogaster*; see the Supplemental Information). One post was omitted in one corner, to make it possible to unambiguously orient the dish.

The resulting microwells for cricket embryos are shown in Fig. 1E, alongside example microwells for two additional species, the three-banded panther worm *Hofstenia miamia* (Fig. 1F) and *E. coqui* (Fig. 1G). Note that in the latter two cases, the mold microwells were not simple rectangles, but instead had more complex shapes. Since the mold inserts are designed in 2D using a simple drawing program (see Supplemental Materials S1), it is easy for a user without prior experience to design and iterate a complex custom mold. Details for all 14 user-tested mold inserts are in Supplemental Materials S1.

With the mold insert ready, we cut and assembled the non-customized OMMAwell components. Detailed assembly instructions with photo guides are in Supplemental Materials S2; the design file for each component is included in Drawing Exchange Format (DXF) and Portable Document Format (PDF) in Supplemental Data. These files can be opened and edited by many design or drawing software packages, including AutoCAD, FreeCAD, Solidworks, SketchUp Pro, Adobe Illustrator, and CorelDRAW. All of the pieces can be made from a single sheet of 6 mm thick acrylic sheet on a laser cutter, which is how we fabricated them for testing. Another option is to 3D print the components by using the included design files as the basis for a 3D model of each piece. If the user does not have access to a laser cutter or 3D printer, pieces can also be fabricated by a variety of online providers.

**Fig. 2.**
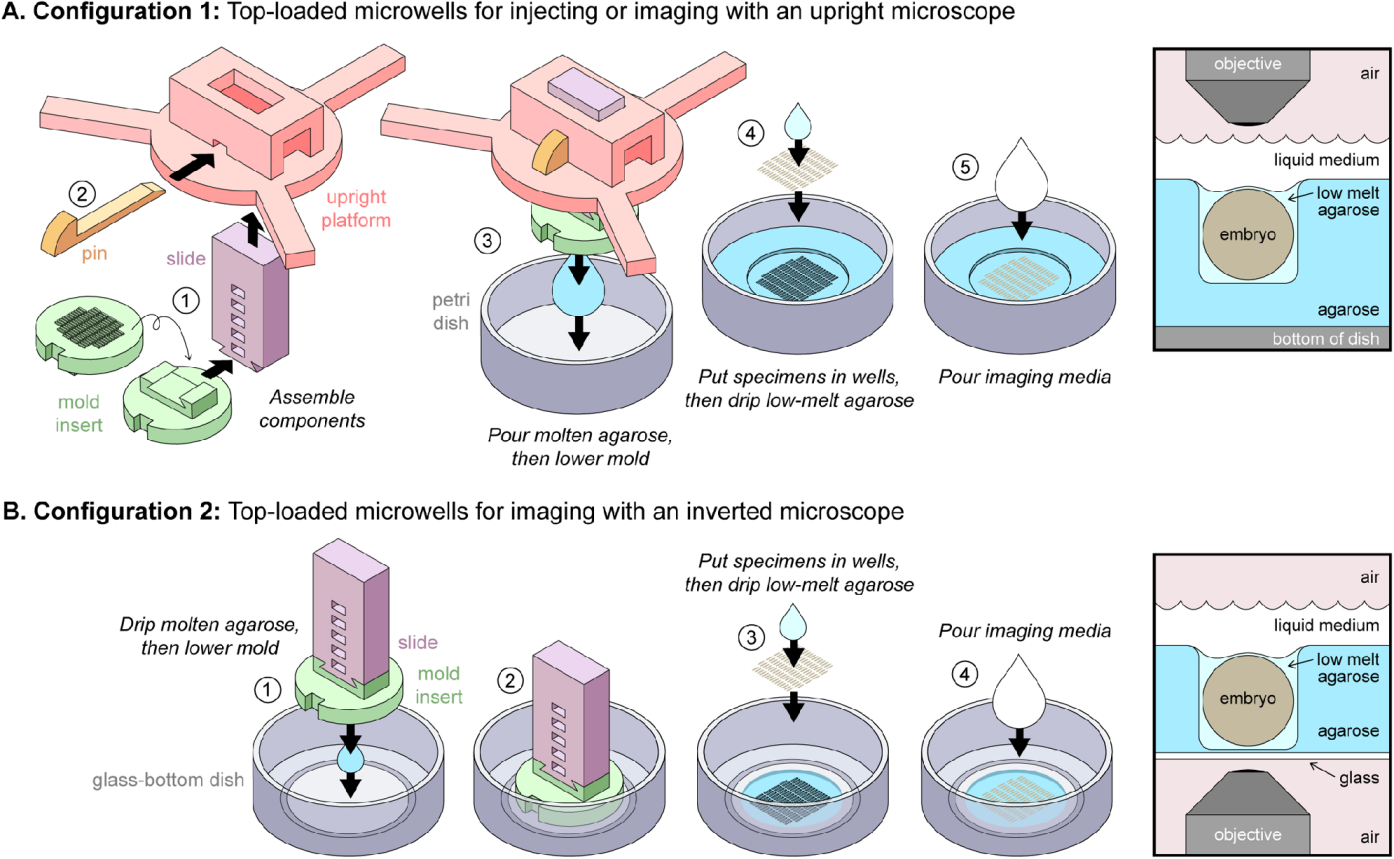
**OMMAwell configurations** **for top-loaded microwells.** (A) Configuration 1: Top loaded microwells for injecting or imaging with an upright microscope. (1) The mold insert (green) is inverted and connected to the slide (purple), which is placed into the upright platform (pink). (2) After the desired height is chosen, the pin (orange) is inserted. (3) Molten agarose is poured into a plastic petri dish and the mold assembly is lowered into it. (4) After the agarose sets, the mold insert is removed and eggs are placed into the wells, either individually with forceps or many at once by transferring the eggs in water with a cut plastic pipette. Excess water is removed by pipet and then wicked away with piece of lint-free lens paper. Then, 40–100 µl of molten low-melt agarose, kept at 42°C, is added to the wells to hold the eggs in place. Embryo positions are adjusted with plastic forceps. (5) When the low-melt agarose sets, the live-imaging medium is added to the dish. Right: Schematic of embryo in Configuration 1. (B) Configuration 2: Top loaded microwells for imaging with an inverted microscope. (1) 700 µl of agarose is pipetted into the middle of the glass-bottom dish. The insert and slide are lowered onto it, taking care not to trap bubbles. (2) Agarose sets, and then the insert and slide are gently removed. (3) Embryos are loaded into microwells, as described above. (4) Live-imaging medium is added. Right: Schematic of embryo in Configuration 2.

**Fig. 3.**
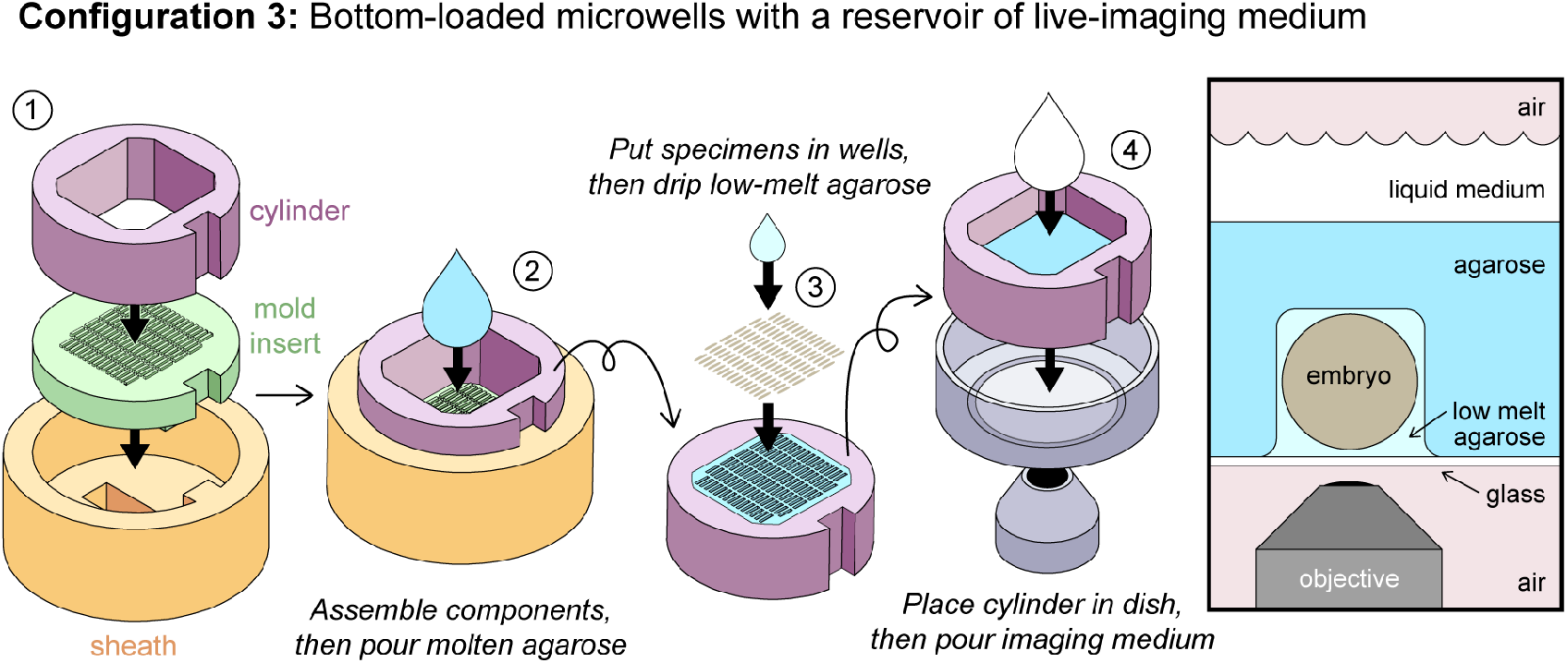
**OMMAwell configurations for bottom-loaded microwells.** Configuration 3: Bottom loaded microwells with a reservoir of live-imaging medium. (1) The insert (green) and cylinder (purple) are placed into the sheath (orange). (2) Molten agarose is poured into the cylinder to the desired depth. (3) Once the agarose has set, the cylinder and the agarose block are removed from the sheath and insert. The cylinder is flipped over, and the exposed microwells are loaded with embryos, as described in Fig. 2. (4) The cylinder and agarose block are lowered into the glass-bottom dish, and live-imaging medium is added in the cylinder. Right: Schematic of embryo in Configuration 3.

For a single mold insert, there are three possible OMMAwell configurations, each of which is useful for different purposes (Figs 2 and 3). Below we discuss the use of each configuration separately.

Configuration 1: Top loaded microwells for injecting or imaging with an upright microscope (Fig. 2A; see legend for step-by-step usage instructions)*.* In this arrangement, the user can adjust the height of the mold insert, which is then lowered into molten 1.5% (w/v) agarose. Once the agarose has cooled and set, the mold is removed, leaving microwells in which to place the samples. Optionally, a small quantity of 0.7% (w/v) low-melt agarose (40–100 µl) can then be added to hold samples in the wells. When they are fixed in place, the live-imaging medium is added. This configuration is well-suited for dipping microscope objectives. It is also useful for holding samples that will be injected, such as with double-stranded RNA, small molecule activators or inhibitors, or recombinant protein (Donoughe et al., 2014). In this configuration, cricket embryos will successfully complete embryogenesis (∼12 days), so long as the medium level is maintained. A drawback of this configuration is that if the working distance of the microscope objective is too short, a lid cannot be added to prevent evaporation. Configurations 2 (Fig. 2B) and 3 (Fig. 3) do not have this drawback.

Configuration 2: Top loaded microwells for imaging with an inverted microscope (Fig. 2B; see legend for step-by-step usage instructions)*.* This is similar to Configuration 1, but the mold insert is placed flat on molten 1.5% (w/v) agarose in a glass-bottom dish, producing microwells in a thin agarose film. The samples are loaded into the wells, fixed in place with 0.7% (w/v) low-melt agarose as above, the dish is covered with its lid, and then imaged from below on an inverted microscope. Because the lid remains on the dish and reduces evaporation, this configuration is the best one for long-term live imaging. As with Configuration 1, but without the need to maintain the medium level manually, cricket embryos mounted in Configuration 2 will develop normally for 12 straight days, completing embryogenesis at rates comparable to unmounted embryos (92–100% hatching). A minor difficulty of this configuration may be that removing the mold insert and slide (Fig. 2B, Step 2) without disrupting the thin agarose film can be a delicate procedure for some insert designs. To ameliorate this problem, we recommend the use of 2% (w/v) agarose to make the microwells. When it has set, pull up the mold insert with the agarose still adhered to it. Then, using plastic forceps, peel the agarose from the insert, return it to the glass-bottom dish, and ‘glue’ it in place with ∼100 µl of 0.7% low-melt agarose. It can take first-time users some practice to become effective in peeling the agarose from the insert, but once it has been peeled, we do not notice any non-uniformities in the wells. Since this configuration relies more strongly than the others on manual manipulation, this technique has more opportunities for variance than the others. In our hands, however, it is a trade-off that can be worth making for some experiments.

Configuration 3: Bottom-loaded microwells with a reservoir of live-imaging medium (Fig. 3; see legend for step-by-step usage instructions). This configuration is recommended for cases where making the agarose film in Configuration 2 is troublesome for a particularly complex mold insert, or if it is necessary to use a larger volume of imaging medium than can be poured into the glass-bottom dish. The main advantage over Configuration 2 is that the mounting process is extremely robust. The downside is that the samples are separated from the imaging medium by a much thicker layer of agarose, which means that gas exchange is reduced. In our hands, cricket embryos mounted in this fashion will typically develop normally for only 6–12 h and then arrest. If the embryos are subsequently removed from their microwells and immersed in water, development continues normally. This configuration also offers a larger reservoir that can be filled and capped with a lid; its volume can be increased further by adding more layers to the ‘cylinder’ in Step 5 of Supplemental Materials S2.

**Fig. 4.**
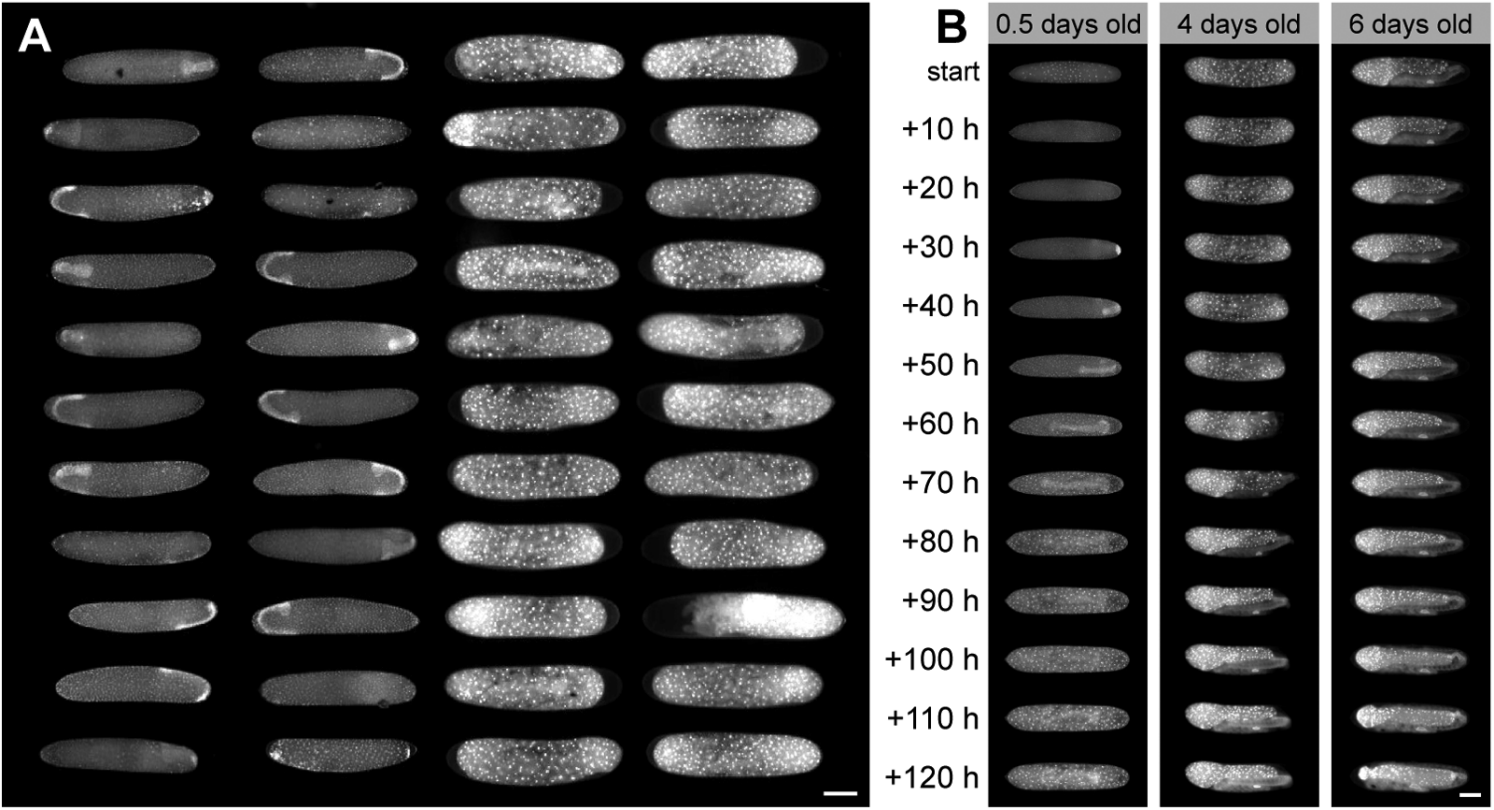
**Arrays of live-imaged embryos within the OMMAwell mold.** (A) A single timepoint from a time-lapse of an array of nuclear-marked transgenic cricket embryos in microwells. The two leftmost columns show germ band stage embryos that are beginning the physical re-orientation within the egg called anatrepsis. The two rightmost columns show a later stage when embryos are fully immersed in the yolk below the extraembryonic membrane called the serosa. (B) Time series of cricket embryos starting at different ages. The ambient temperature is ∼24°C, so development is slower than that reported by Donoughe and Extavour (2016). Scale bars: 500 µm.

We have used each of these three configurations (Figs 2 and 3) to live-image more than 100 embryos simultaneously. In some species, embryonic development may be particularly sensitive to oxygen supply. If this is a concern, Configuration 2 is most suitable, as it minimizes the amount of agarose around the embryos. For our work with crickets, we can oxygenate embryos by manually bubbling and stirring the imaging media, although this is not required for healthy development. An automated approach for oxygenation would require additional tool development that we have not explored. For species with smaller embryos, the maximum sample size is even larger, and it is up to the user to determine the desired density and number of wells. If the array of wells is larger than the microscope's field of view, the user can either manually move the stage or use a motorized stage to move the array in the X-Y plane so that all the samples can be imaged. Because each well has a unique identifier, even with a manual approach, large numbers of individual embryos can be followed and uniquely identified over time-lapse periods. As an example, we show a single time point from a time-lapse of 44 nuclear-marked transgenic cricket embryos (Fig. 4A). We used a motorized stage to capture tiled micrographs of the full set of eggs once every 5 min. The recording continued for 5 days with no signs of phototoxicity or developmental defects. The specimens were then returned to the incubator, and 41 of the 44 embryos hatched, survivorship that is comparable to embryos that were not mounted or imaged (this ranges from 90–100% across trials). We do not observe developmental delays in imaged embryos. In the case of crickets, we routinely transfer embryos from the agarose wells to another dish, mid-development, with no loss in embryo integrity. We have not systematically tested survivorship following this procedure with other species. For species with fragile embryos, the question of whether or not such a transfer procedure might be possible without unacceptably compromising survivorship would have to be determined empirically.

Researchers can easily design and fabricate their own mold inserts to generate wells of specified shapes, dimensions, and spacing. Patterns can be designed *de novo*, or altered from the insert files included with this article (Supplemental Data). If the user fabricates a piece from acrylic using a laser cutter, the design can be simply made as a 2D form, like those described in Supplemental Materials S1. If the user prefers to a use a 3D printer, the included design files can be used as the basis for creating a new 3D design file. A brief comparison of the advantages and disadvantages of each mode of fabrication is given in Supplemental Materials S3.

## Materials and Methods

Design and assembly details are given in Supplemental Materials S1 and S2. When making the microwells, agarose (Bio-Rad #1613101) was dissolved at 1.5% weight/volume (w/v) in distilled water (or 2% for firmer molds). Then, eggs were held in microwells with low-melt agarose (Bio-Rad #1613112) at 0.7% (w/v) in distilled water. Tap water was used as a live-culturing solution for wild-type cricket eggs, but the user could also pour molds with agarose dissolved in a live-imaging buffer that is appropriate for their samples.

*G. bimaculatus* wild-type strain was originally reared in Yamagata prefecture, Japan. Wild-type eggs were imaged with transmitted white light on a Zeiss Lumar dissection microscope. For fluorescent imaging, recordings were taken of eggs from a transgenic line in which the cricket actin promoter drives expression of the cricket Histone-2B protein fused to Enhanced Green Fluorescent Protein (H2B-EGFP) (Nakamura et al., 2010). The 5× objective on a Zeiss Celldiscoverer 7 was used for imaging. We have also successfully imaged OMMAwell-mounted embryos with 10× and 20× objectives, but we have not systematically tested all mounting options on these and other higher magnification objectives. In each case, success or failure will depend on the working distance of the objective, the size of the specimens, and which of the three OMMAwell configurations is being used. The array of microwells was tiled with the motorized stage under the control of Zen software (Zeiss, Oberkochen, Germany). A z-stack was captured at each position, then later combined using Zen's ‘Extended Depth of Focus’ (mode=‘Contrast’). Figures were prepared using Illustrator (Adobe).

## Supplementary Material

Supplementary information
