# Supplementary material for: High-throughput live-imaging of embryos in microwell arrays using a modular specimen mounting system
Source: Biol Open. 2018 Apr 30;7(7):bio031260. doi: 10.1242/bio.031260 (PMC6078342; doi:10.1242/bio.031260)
Supplement: Supplementary information [file biolopen-7-031260-s1.pdf]

Supplemental materials S1: Design files for user-tested mold inserts

Design files for user-tested mold inserts

This document contains descriptions and schematics for 14 mold inserts that were designed to be used for a range of study organisms, including zebrafish, fruit fly, mouse, frog, annelid worm, amphipod, red flour beetle, acoel, and cricket. Each insert is compatible with the adjustable-height mounting tripod described elsewhere in this paper.

Components were designed using FreeCAD and Adobe Illustrator. Each mold component was made by cutting it from a sheet of 6.35 mm optically clear extruded acrylic (McMaster-Carr #8560k355). A laser cutter (Universal PLS 6.75) in engraving mode was used to generate mold inserts by removing acrylic in a pattern, leaving a grid of raised posts. When pieces needed to be combined, they were fused together in pairs using acrylic welding solvent (Weld-on 3 Assembly Adhesive, SciGrip #10799) (see Supplemental Materials S3 for details). Most of these mold inserts were designed to fit within a 35 mm diameter plastic petri dish with a 27 mm diameter glass coverslip bottom (VWR #89428-990). Many microscopes need a stage insert in order to stably hold a petri dish (e.g. Zeiss #432311-9901-000). In Configuration 1, the device can be used on a range of dish sizes; we tested it on 6 cm (VWR #25384-092) and 10 cm (VWR #25384-342) diameter plastic petri dishes.

To use these inserts with the OMMAwell device, simply attach slots to the back of the insert, following the procedure in step #7 of the assembly instructions in Supplemental Materials S3. Details for each insert are included below. Each schematic has been magnified for presentation and the key dimensions are given with each insert. For complete dimensions, please see the design files in Supplemental Materials S2. These are included in two formats: PDF and DXF. PDF files are readily viewed and edited by many common software packages. DXF is a file type that can be opened in a wide variety of drafting and vector graphics software packages, including AutoCAD, FreeCAD, Solidworks, SketchUp Pro, Adobe Illustrator, and CorelDRAW. Unlike PDF, DXF files contain precise length information. We hope readers will use these as a starting point to make their own inserts; if so we would be happy to hear about it. Each of these mold inserts can be made from 6 mm-thick acrylic sheet using a laser cutter. Alternatively, if a user prefers to fabricate them with a 3D printer, the user can adapt the included design files to make a 3D model of each component. The depth dimensions of each part is listed below. See Supplemental Materials S4 for a brief comparison of the two approaches.

Identifying wells: For some molds, there are symbols or numbers to identify columns or rows on the agarose wells. These provide the "coordinate address" of each well. In other molds, the arrangement of the molds themselves – that is, an arrangement without rotational symmetry – is sufficient information to uniquely identify each well.

Note: At the end of this document is a section that discusses considerations for using wells to hold specimens for injection.

Capitella teleta, 100-well

Insert_Capitella_Injection_200x200um_100n

Description: This mold layout was designed in collaboration Dr. Mark Martindale (University of Florida), Dr. Elaine Seaver (University of Florida), and Dr. Mansi Srivastava (Harvard University), for injecting the spherical eggs of marine invertebrates and acoel worms. Eggs are placed into the square wells and then injected with a pulled glass needle entering from the right. When an egg gets stuck on the needle, the user can move the needle tip into the large well on the left, and then slide the needle up and out through one of the thin troughs, freeing the egg from the needle. This mold been tested with eggs from the marine annelid worm *Capitella teleta*, but by adjusting the dimensions of the square microwells, this mold insert can easily by adapted to a wide range of species. To make this mold, cut on the **red** line, etch the **cyan** region to a depth of ~800 µm, and etch the **orange** regions to a depth of ~550 µm.

Dimensions: This insert generates agarose microwells that are 200 µm square, with a depth of 250 µm. The large well on the left has a depth of 800 µm. The outer margin of the insert is a 31 mm diameter circle to fit in a 35 x 10 mm plastic dish (Bio-One #627160) for injection.

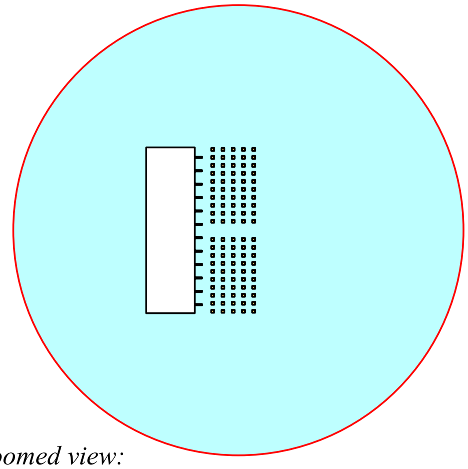

Zoomed view:

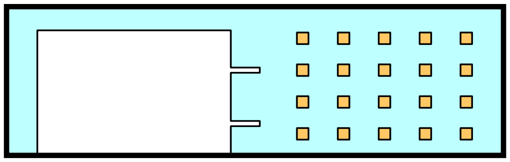

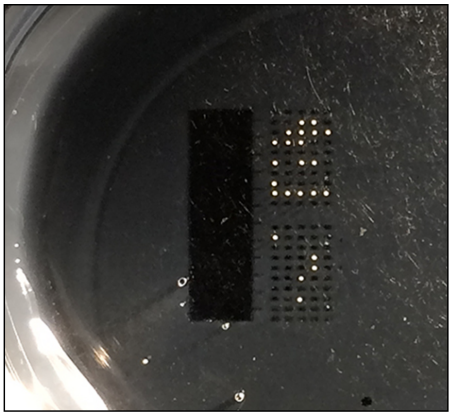

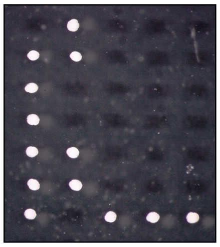

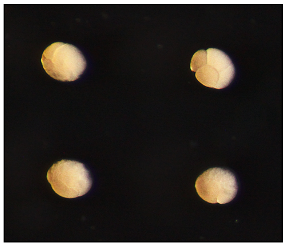

Photos by Elaine Seaver (University of Florida)

Biology Open • Supplementary information

Danio rerio, chorions intact, 96-well

Insert_Danio_1100x1100um_96n

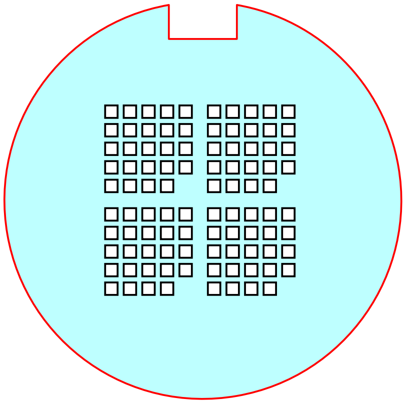

Description: This mold was designed for injecting and live-imaging zebrafish embryos with their chorion intact. To make this mold, cut on the **red** line, then etch the **cyan** region to a depth of ~1300 µm.

Dimensions: Each microwell is 1100 µm square, with a depth of 1100 µm. The outer margin of the insert is a circle with radius 35 mm to fit snugly into a glass-bottom dish for live-imaging with an inverted microscope and into the mold sheath (see Supplemental Materials 3). The wells are placed so that the embryos all land within the 27 mm diameter of the glass coverslip bottom (VWR #89428-990). The same is true for all mold inserts described in this document that have a rectangular notch.

Danio rerio, chorions removed, 96-well

Insert_Danio_800x800um_96n

Description: This mold is used for live-imaging zebrafish embryos whose chorions have been removed. To make this mold, cut on the **red** line, then etch the **cyan** region to a depth of ~800 µm.

Dimensions: Each microwell is 800 µm square, with a depth of 1000 µm. The outer margin of the insert is a circle with radius 35 mm. It is compatible with the glass-bottom dishes and the mold sheath, as described above.

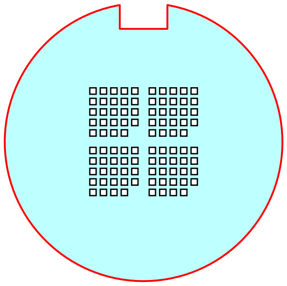

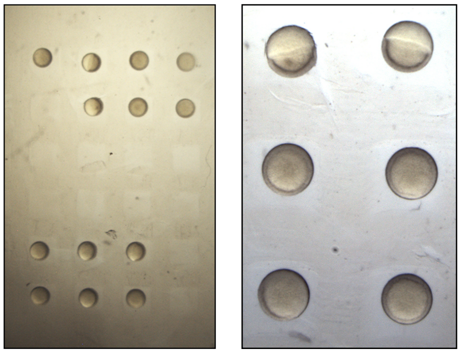

Photos by Megan Norris (Harvard University)

Danio rerio, embryos with growing tails, 47-well

Insert_Danio_Lateral_Wells_47n

Description: During embryogenesis, the growing tail of zebrafish embryos makes it difficult to keep them in place during long term live-imaging. To address this problem, this mold generates circular wells for the spherical yolks to settle into, and adjacent troughs for the elongating tails to enter. To make this mold, cut on the **red** line, etch the **cyan** region to a depth of ~500 µm, and etch the **orange** regions to a depth of ~250 µm. *Note:* at these depths, the embryos's midline is approximately flush with the upper surface of the agarose, meaning that the larva protrudes upwards, making this mold best suited for imaging on an upright microscope, but if the etchings were 2-fold deeper, it would be suitable for inverted microscopy as well.

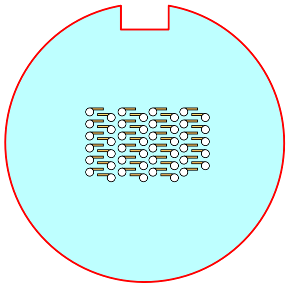

Zoomed view:

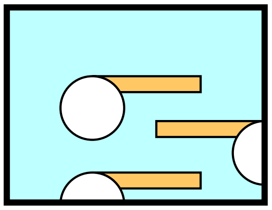

Dimensions: The circular portion of each microwell is 1 mm in diameter, with a depth of 0.5 mm. The tail trough is 1.7 mm long, 0.25 mm wide, and 0.25 mm deep. This mold is compatible with the mold sheath and the aforementioned glass-bottom dishes.

Danio rerio, injection troughs

Insert_Danio_Injection_Troughs_10n

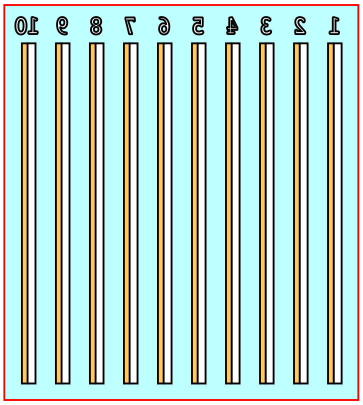

Description: This mold makes troughs that can hold many zebrafish eggs for injection. Each trough has a stepped notch removed from one side so that the needle can enter the eggs without getting stuck in the agarose. To make this mold, cut on the **red** line, etch the **cyan** region to a depth of ~700 µm, and etch the **orange** regions to a depth of ~350 µm.

Dimensions: Each trough is 30 mm long and 0.7 mm deep at the bottom. A trough is 0.7 mm wide at the bottom, and 1.2 mm wide at the top. The outer margin of the insert is a rectangle 31.2 by 34.8 mm. This insert will not fit in the mold sheath, but once slots are attached to the back, it can be used with the adjustable-height tripod.

Drosophila melanogaster, 294-well

Insert_Drosophila_220x500um_294n

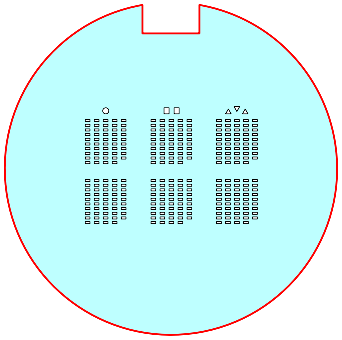

Description: This mold was designed for live-imaging arrays of fruit fly embryos. The etched shapes make it easier to navigate the array of microwells while using a microscope. To make this mold, cut on the **red** line, then etch the **cyan** region to a depth of ~240 µm.

Dimensions: Each microwell is 500 µm long, 220 µm wide, and 240 µm deep. The outer margin of the insert is a circle with radius 35 mm. It is compatible with glass-bottom dishes and the mold sheath.

Eleutherodactylus coqui, 24-well

Insert_Eleutherodactylus_4200x4200um_24n

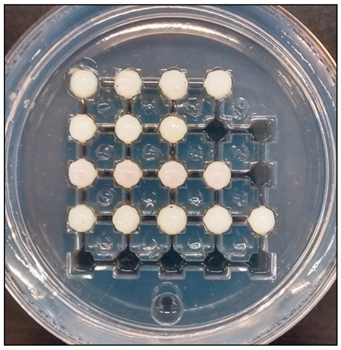

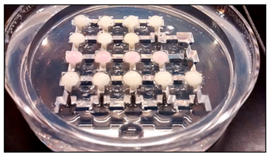

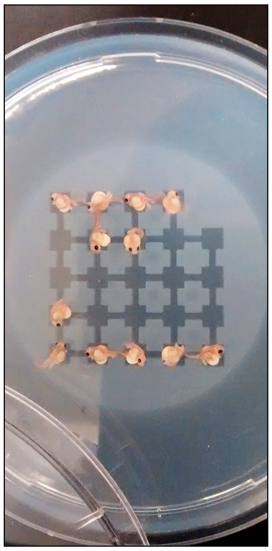

Description: This mold insert was designed to produce wells for injecting and live-imaging the embryos and larvae of the coquí frog. The channels in the agarose admit the tails of the larval frogs so that they can more easily be oriented by the user for microscopy. To make this mold, cut on **red** lines. Then use acrylic solvent (see Supplemental Materials S3 for details) or cyanoacrylate glue to attach the two pieces flat together, lining up the circular holes on the circular piece with the plus-sign-shaped holes on the rectangular piece. The holes allow air to enter and release the vacuum when removing the mold from the agarose.

Dimensions: Each well is 4.2 mm square; the channels connecting the wells are 1.5 mm wide. The array of wells is a square 32.2 mm to a side. The circular baseplate has a diameter of 47.8 mm. By varying the amount of agarose poured the user can adjust the depth of the wells.

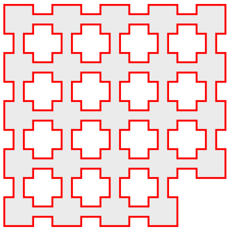

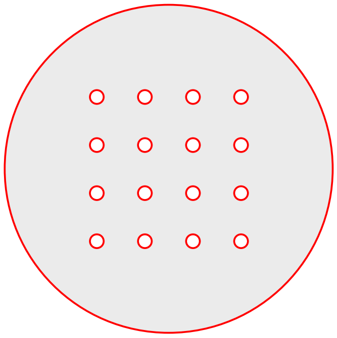

Photos by Mara Laslo (Harvard University)

Gryllus bimaculatus, 120-well

`Insert_Gryllus_570x2930um_120n`

Description: This mold insert was designed to fit the maximum number of cricket embryos on a single glass-bottom dish. Cut on **red** lines, and etch the **cyan** region to a depth of ~650µm.

Dimensions: Each microwell is 2930µm long, 570µm wide, and 650 µm deep. The outer margin of the insert is a circle with radius 35 mm. It is compatible with glass-bottom dishes and the mold sheath.

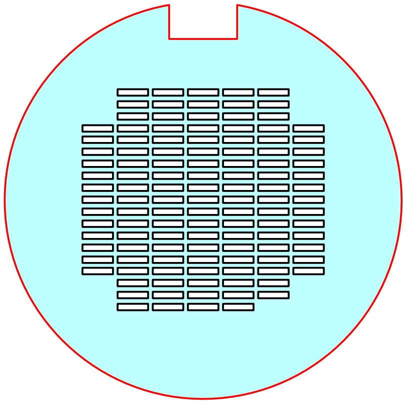

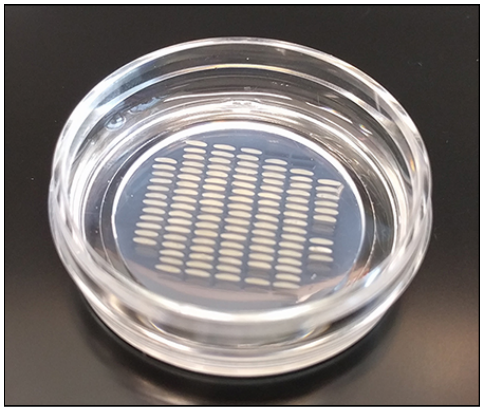

Gryllus bimaculatus, 66-well

`Insert_Gryllus_570x2930um_66n`

Description: This is similar to the previous mold insert, but with fewer wells and etched shapes to aid in the orientation on the microscope. Wells are arranged into a rectangle for time-efficient automated tiling. Cut on **red** lines, and etch the **cyan** region to a depth of ~650µm.

Dimensions: Same dimensions as previous insert.

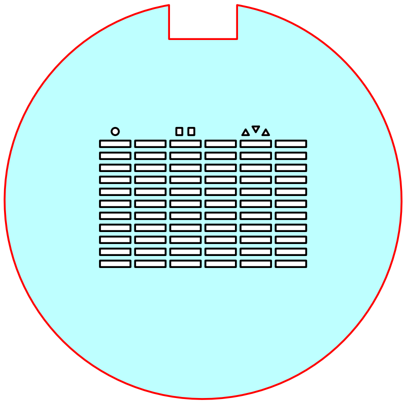

Gryllus bimaculatus, 300-well

`Insert_Gryllus_Injection_570x2930um_300n`

Description: This mold insert is used for injecting large numbers of cricket eggs at once. Wells are arranged in groups of ten and labeled with an etched number. Cut on **red** lines, and etch the **cyan** region to a depth of ~750µm.

Dimensions: Each microwell is 2930µm long, 570µm wide, and 750 µm deep. Depending on the user's microinjection apparatus it may be helpful to adjust the depth of the wells.

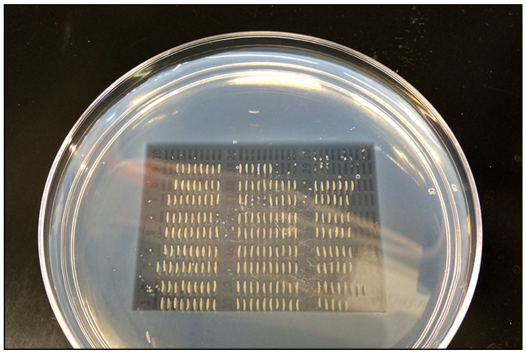

Photo by Taro Nakamura (Harvard University)

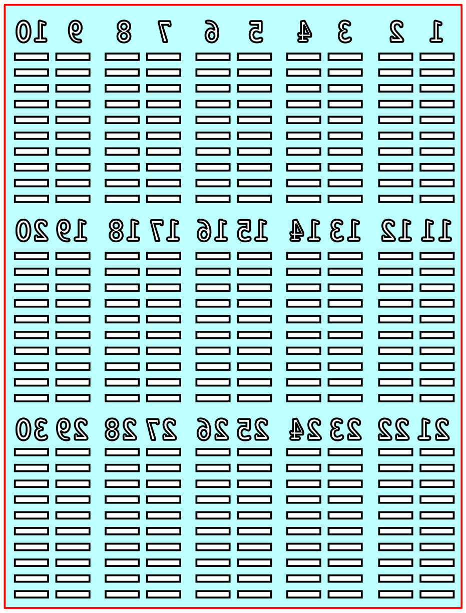

Biology Open • Supplementary information

Hofstenia miamia, 100-well

Insert_Hofstenia_Injection_275x275um_100n

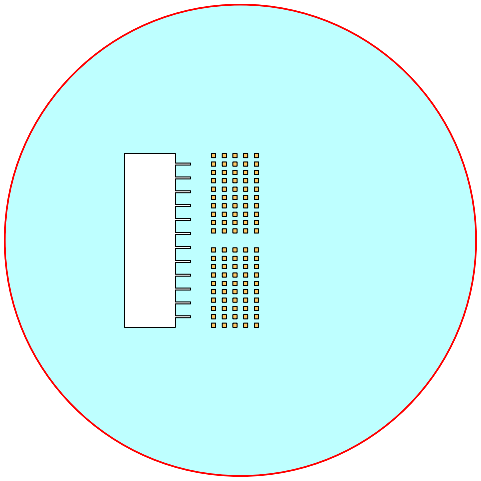

Description: This mold insert is quite similar to the *Capitella teleta* insert described at the beginning of this document; the only difference is that the wells are larger to fit the eggs of the acoel worm *Hofstenia miamia*. To make this mold, cut on the **red** line, etch the **cyan** region to a depth of ~800 µm, and etch the **orange** regions to a depth of ~500 µm.

Dimensions: The insert generates agarose microwells that are 275 µm square, with a depth of 300 µm. The large well on the left has a depth of 800 µm. The outer margin of the insert is a circle with radius 31 mm to fit in a 35 x 10 mm plastic dish (Bio-One #627160) for injection.

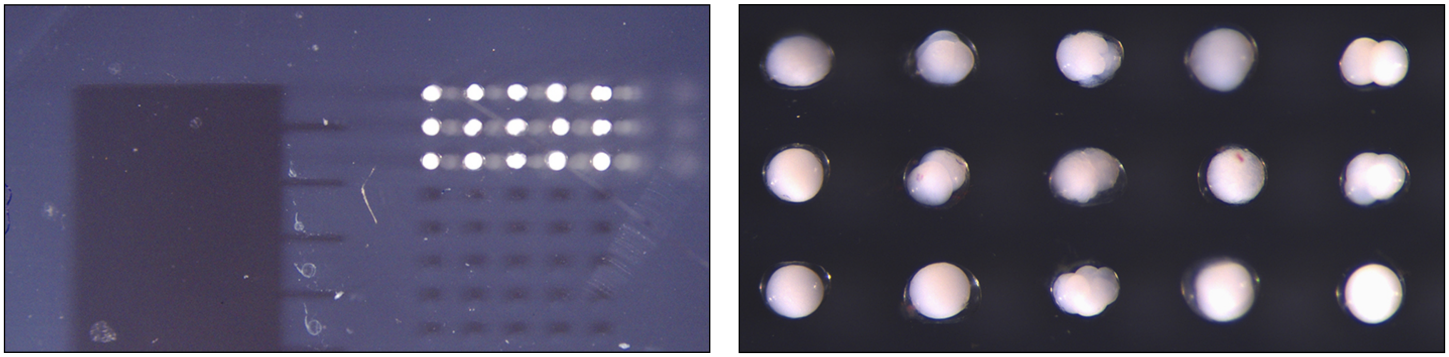

Photos by Andrew Gehrke (Harvard University)

Mus musculus, neurospheres, 101-well

Insert_Mus_Neurospheres_1000x1000um_101n

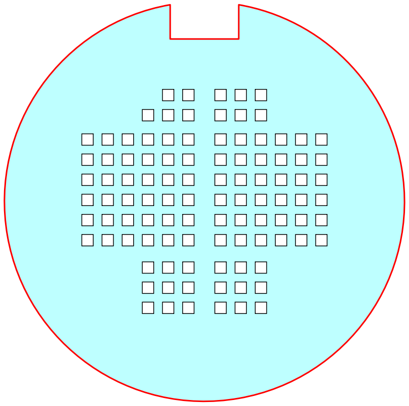

Description: Neurospheres are clusters of cultured cells derived from neural stem cells. This mold insert was designed to make wells to hold neurospheres for imaging. Cut on **red** lines, and etch the **cyan** region to a depth of ~1000µm.

Dimensions: Each microwell is a cube 1 mm to a side. The insert is compatible with glass-bottom dishes and the mold sheath.

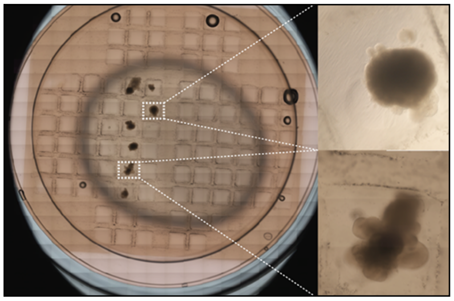

Photos by Richard Smith (Harvard Medical School)

Parhyale hawaiensis, 396-well

Insert_Parhyale_round250um_396n

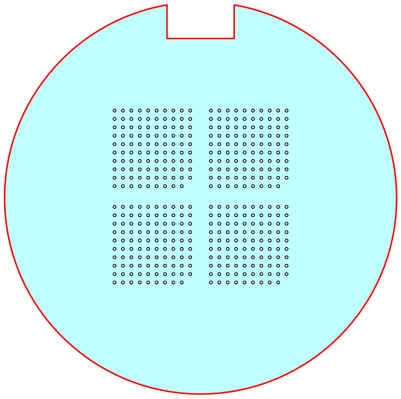

Description: *Parhyale hawaiensis* is a marine amphipod crustacean, an emerging model system for the study of regeneration, germ cell specification, and limb differentiation. This mold insert was designed to make wells to hold its spherical embryos for injection and live-imaging. Cut on **red** lines, and etch the **cyan** region to a depth of ~300 µm.

Dimensions: Each microwell is 250 µm long, 250 µm wide, and 300 µm deep. The insert is compatible with glass-bottom dishes and the mold sheath.

Biology Open • Supplementary information

Tribolium castaneum, 147-well

Insert_Tribolium_350x600um_147n

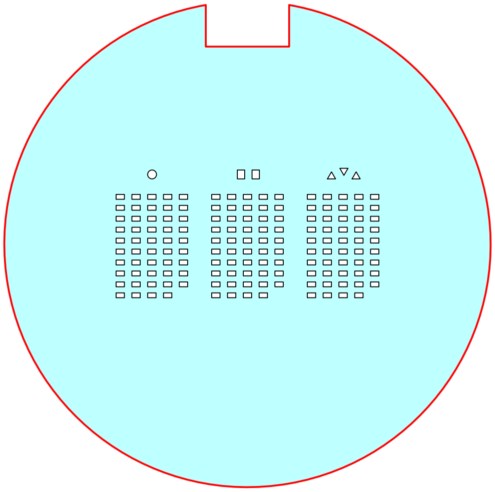

Description: The red flour beetle is a pest of stored grain products and an emerging model system for a range of animal developmental processes. This mold was designed to produce wells that fit the embryos for timelapse live-imaging. Cut on **red** lines, and etch the **cyan** region to a depth of ~375 µm.

Dimensions: Each microwell is 600 µm long, 350 µm wide, and 375 µm deep. The insert is compatible with glass-bottom dishes and the mold sheath.

Considerations for using wells to hold specimens for injection

Several of the molds listed here have been used for successful injections, including the molds for zebrafish, cricket, coqui frog, three-banded panther worm, and annelid worm. When users test injection protocols for additional organisms, these molds and the usage described above my be helpful, but every species will have idiosyncratic challenges.

For instance, some embryos increase in size and/or become motile during development, which might necessitate a transfer from the injection wells to another vessel for subsequent development. Such transfers work effectively in crickets, amphipods, and coqui frogs. We have not tested mid-embryogenesis transfers for other species. Another potential concern is that eggs of some species might rotate under the injection needle. One possible solution for this is to adjust the shape of the well so that the egg can be jammed into a corner during injection.

Some users have also found it helpful to adjust the osmolarity of the acqueous medium in order to increase (or decrease) the swelling of specimens in the wells during injection.

Biology Open • Supplementary information

Supplemental materials S2: Guide for assembling OMMAwell

Guide for assembling OMMAwell

Materials

- Extruded acrylic, 6.00 to 6.35 mm thick (see note below)
- Acrylic welding solvent

Notes on acrylic: Some suppliers sell "¼ inch thick extruded acrylic," while others sell "6 mm thick acrylic." In each case, the actual measured thickness of the material that is delivered can vary from 6.00 to 6.35 mm. We thus designed the components to fit together for any acrylic thickness from 6.00 to 6.3 mm. The pieces will fit a little more loosely with 6.00 mm acrylic and more snugly with 6.35 mm acrylic, but we've tested the designs successfully with both. Another consideration is that opaque / pigmented acrylic is often autofluorescent. Thus for any component that will be mounted in a microscope for fluorescent imaging (namel , the cylinder), it is important to cut the parts out of optically clear acrylic (such as McMaster-Carr #8560k355), although for illustration purposes the photos in this guide depict opaque white acrylic.

Equipment

We used a Universal Laser Systems laser cutter, model PLS 6.75 (panel E below). Any laser cutter/engraver that can cut acrylic of this thickness will do.

How to fuse acrylic components together

Fuse pieces together by first arranging them in the desired configuration (you can use masking tape to hold them together while fusing, although in our hands this was not necessary). Then drip acrylic welding solvent (e.g. IPS Weld-On 3 Acrylic Plastic Cement) into the joint with the supplied applicator or a polypropylene syringe with a blunted steel tip (A, B). The solvent has a very low viscosity and will easily wick into tight joints. It is not necessay to apply any pressure. When connecting two pieces that share a flat surface, another option is to apply a few drops to the flat surface of one piece, and then place the second piece on top (C, D). Assemble pieces on a disposable surface or on a piece of glass. Extra solvent will evaporate away in seconds. Pieces will be fixed in place within a minute, and fully set within a few hours.

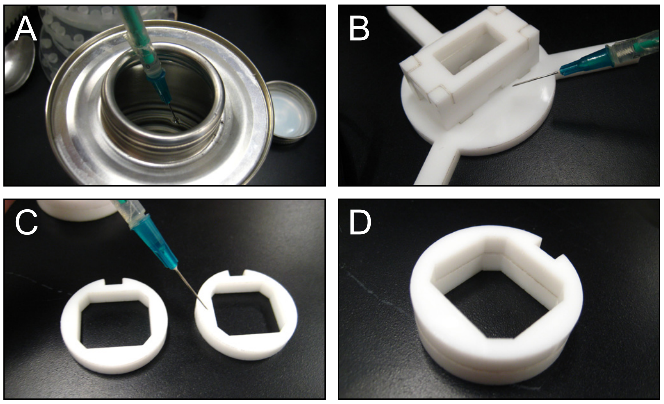

Step-by-step instructions

1) *Laser cut the acrylic pieces.* In Supplemental Materials S3, the file named "OMM well_all_components.dxf" has all the pieces except for the insert. The reader could use one of the user-tested inserts described in Supplemental Materials S2, or design a custom insert. Most acrylic comes with a protective backing. Leave it on during cutting (E, F, G).

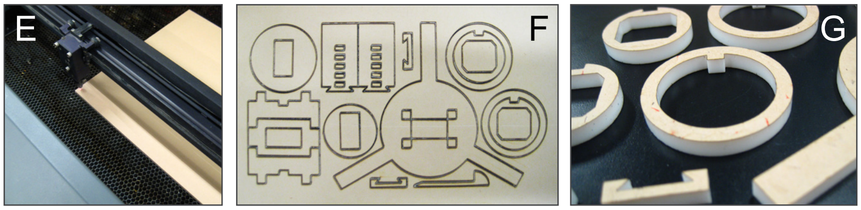

2) *Peel the backing from all pieces* (H). The remaining steps walk through how to fuse the acrylic parts together for each component.

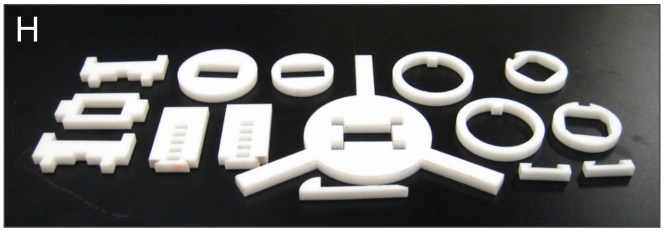

3) *Assemble the upright platform.* Gather the 4 pieces shown (I), then slot the side walls into the tripod and fuse them in place (J). Then add the top slot and fuse in place (K). Set aside.

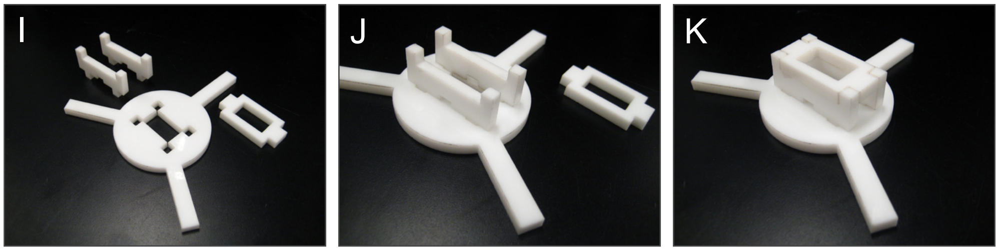

Biology Open • Supplementary information

4) *Assemble the slide*. Take the two identical pieces, align them on top of one another, and then fuse them together (M, N).

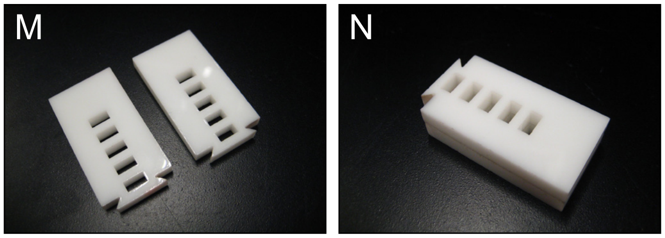

5) *Assemble the cylinder*. Take the two identical pieces, align them on top of one another, and then fuse them together (O, P).

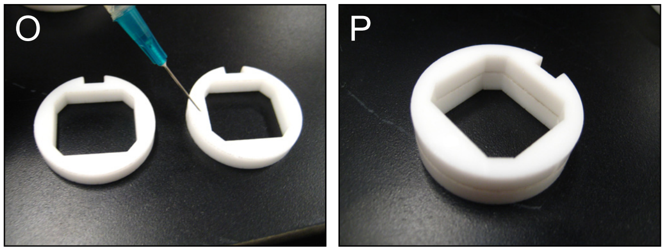

6) *Assemble the sheath*. Take the two identical ring pieces (Q), align them on top of one another, and then fuse them together. Then fuse these to the base as shown at right, with the protruding rectangular tab centered on the short end of the rectangular hole in the base (R, S).

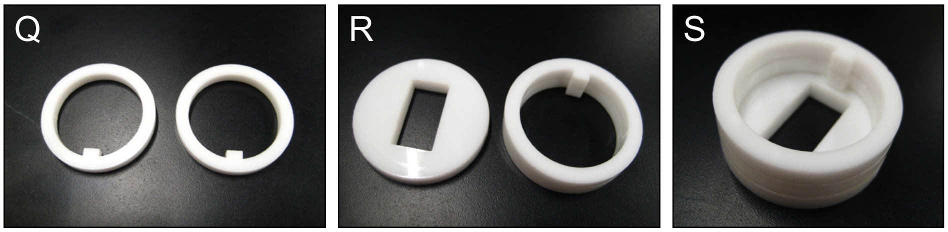

7) *Attach the slots to the back of an insert*. Once you have designed and fabricated an insert (e.g. T), you will need to attach slot pieces to the back of it. First place the insert into the now-completed sheath (U), and then trace the rectangular opening on its flat side (, W) and set aside the sheath (X). Position and fuse two slot pieces side by side in the traced rectangle (Y).

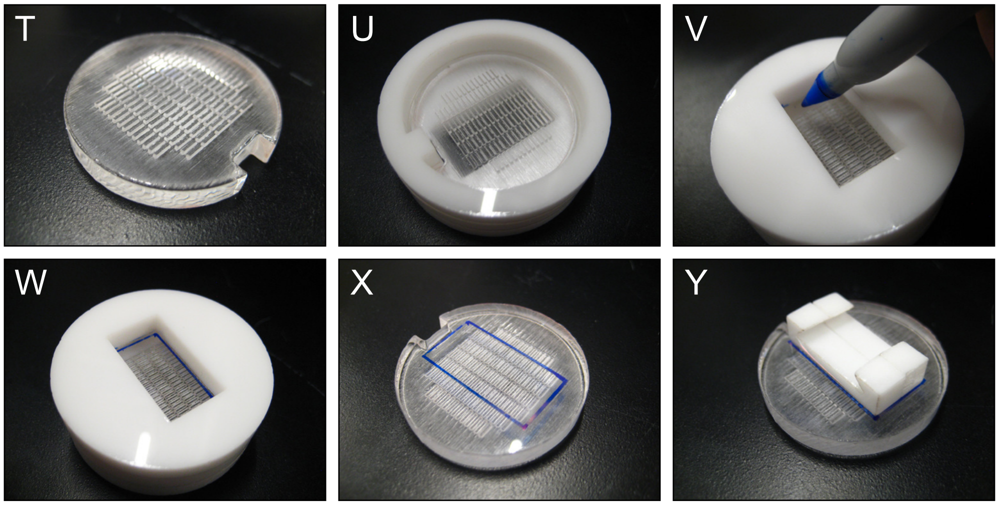

NOTE: The part highlighted in the red box (Z) is an optional piece called the slide lock. When using Configuration 1 (Figure 2, top), this piece can be put on the slide before connecting the insert to the slide. It then locks the insert in place.

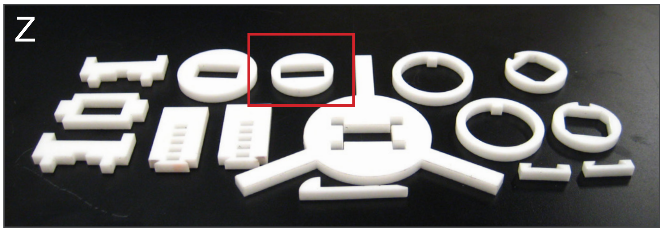

Biology Open • Supplementary information

Supplemental materials S3: Comparison of fabrication techniques

In testing OMMAwell, we fabricated all the components using laser cut acrylic, however 3D printing is also an option. The two modes of fabrication differ in many respects, including 1) Equipment cost, 2) Choice of materials, 3) Cost of materials, 4) Speed of fabrication, 5) Ease of file design. Depending on how a user weighs these, and depending on what they have access to, each user will come to their own conclusion about which is best.

	Equipment cost	Choice of materials	Cost of materials	Speed of fabrication	Ease of file design
Laser cut plastic	Laser cutters and 3D printers vary enormously in sophistication, resolution, and price. A new laser cutter or 3D printer that is capable of fabricating the pieces described in this article could be purchased for a minimum price of 2000-4000 United States Dollars (USD). For our tests we used a PLS6.75 laser cutter. New, this machine would cost about 20,000 USD, but it was much larger and more powerful than was needed to fabricate the components discussed in this article.	In our hands, the easiest and cheapest plastic to use for laser cutting and engraving is acrylic. Acrylic comes in clear or opaque varieties. One drawback of acrylic is that ethanol will cause cracks to form in it, eventually causing components to break down. Thus, for cleaning, it is better to use soap and water. We have not tested other plastics for engraving.	All the OMMAwell components described in Supplemental Materials S3 can be cut from a sheet of 6mm-thick acrylic that is 12 x 20 cm. We purchase acrylic from McMaster-Carr (e.g. Cat# 8560K356). The amount of material to produce all OMMAwell components costs approximately 5 USD.	Laser cutting all the OMMAwell components described in Supplemental Materials S3 takes approximately 30 minutes, plus an additional ~30 minutes to weld together the pieces.<br><br>Engraving a single mold insert takes 10 to 30 minutes, depending on size.	Laser cutting and engraving is generally done as a two-dimensional process. This means that design files can be quickly produced by anyone who can make 2D vector drawings (such as in Adobe Illustrator, Corel Draw, or Inkscape). The downside of this approach is that the complexity of the eventual 3D form is limited to shapes constructed of vertical and horizontal planes.
3D printed plastic		We have not tested 3D printed materials for OMMAwell components, but there is a wide range of options. Wittbrodt et al (2014) printed a mold for zebrafish larvae using polylactic acid (PLA). Gregory and Veeman (2013) ordered a mold for ascidians made from MicroFine Green resin, a material produced by Fineline Prototyping. Alessandri et al (2017) printed components of an imaging chamber using HTM140 resin.	This varies widely, depending on the material and provider.	This will vary depending on the 3D printer that is used, but for components of the scale of OMMAwell, printing duration will likely range from a few hours to overnight.	3D printers will require a fully three-dimensional design file. There are several options for inexpensive or free software tools for this, as well as professional options. Each of these will have a steeper learning curve than those needed for making a 2D design, but the range of possible 3D forms is much larger.

Supplemental Data

Click here to download Supplementary Data S1
